# Chronic Exertional Compartment Syndrome of the Hand With Simultaneous Carpal Tunnel Syndrome and Ulnar Nerve Neuropathy: A Clinical Case Report

**DOI:** 10.7759/cureus.27480

**Published:** 2022-07-30

**Authors:** Sean M Bowling, Nicholas L Todd, Jonathan T Avon, Mallory D Jengo, Micah W Jones

**Affiliations:** 1 Department of Orthopedic Surgery, Edward Via College of Osteopathic Medicine, Blacksburg, USA; 2 Department of Orthopedic Surgery, LewisGale Medical Center, Salem, USA

**Keywords:** vcom, orthopaedic hand surgery, chronic exertional compartment syndrome, ulnar nerve neuropathy, carpal tunnel syndome

## Abstract

Chronic exertional compartment syndrome is a reversible form of compartment syndrome that occurs with exertion and is relieved with rest. Chronic exertional compartment syndrome most commonly occurs in the lower leg and has only rarely been reported in the hand. We report a case of exertional compartment syndrome in the left hand of a 37-year-old male heavy equipment technician with concurrent carpal tunnel syndrome and ulnar neuropathy. Physical examination showed non-exertional numbness and tingling in all five digits while at rest with a reproducible Tinel’s test over the carpal tunnel and Guyon’s canal. Acute swelling and hand muscle weakness appeared after repetitive pinch and usage of the thenar and intrinsic musculature with acute sensory and motor changes in the ulnar nerve distribution. Elective fasciotomies were performed in the first dorsal interosseous and thenar compartments with concomitant release of the carpal tunnel and ulnar nerve at the wrist. The patient exhibited a full recovery from symptoms with no residual functional deficits. Although rare, patients that perform repetitive hand motions can develop chronic exertional compartment syndrome. To our knowledge, this is the first reported case of chronic exertional compartment syndrome in the hand that occurred with chronic overuse neuropathies and an acute ulnar neuropathy with intrinsic hand muscle weakness at the same time. It is important for providers to conduct a thorough history and physical examination to differentiate multiple hand pathologies that may present simultaneously.

## Introduction

Acute compartment syndrome (ACS) occurs when elevated fascial compartment pressure impedes tissue perfusion, leading to muscle necrosis [1]. Chronic exertional compartment syndrome (CECS) is essentially a reversible form of ACS, where symptoms start with exertion and resolve with rest [1]. It is characterized by pain, swelling, and decreased muscle function with exertion [2]. CECS is most commonly diagnosed in the anterior lower leg compartment of long-distance runners and military personnel, but rare cases have been reported in the thighs, forearms, and hands [1]. Hand CECS is rarely reported in the literature, and to our knowledge, no reports demonstrate CECS with median and ulnar neuropathy occurring simultaneously [3]. We present a 37-year-old male diagnosed with CECS in the first dorsal interosseous and thenar compartments with concurrent carpal tunnel syndrome and acute thumb and ulnar nerve neuropathy at the wrist.

## Case presentation

We present a 37-year-old left-hand dominant male who presented to the outpatient orthopedic clinic with complaints of eight months of left-hand pain and numbness in the median nerve distribution as well as swelling in the first dorsal web space. Symptoms worsened with activity and significantly limited his ability to perform his job as a machine technician. He noted that he frequently used drills and screwdrivers at work to install heavy equipment and these activities exacerbated his symptoms. The patient could reproduce symptoms by repetitively squeezing a stress ball. These symptoms included active swelling, acute weakness of his first dorsal interossei and thenar muscles with weak pinch and thumb adduction, and overall hand weakness. All symptoms apart from numbness resolved completely after 20 to 30 minutes of rest.

**Figure 1:**
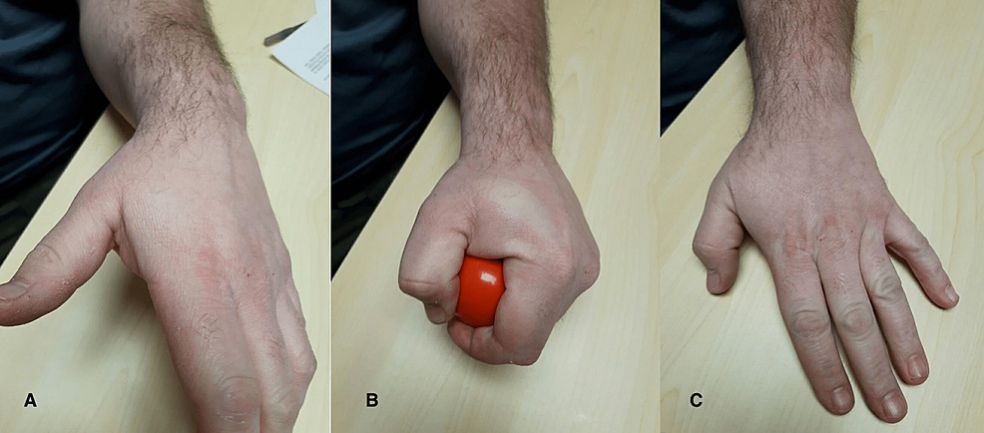
(A) Image before exertion. No acute swelling of the first webspace. (B) Image after exertion. Notice the acute swelling of the first web space with marked pallor of the skin from the increased pressure of the underlying first dorsal interosseous compartment. (C) Image after exertion showing a positive Wartenberg’s sign, indicating acute ulnar nerve weakness and unopposed, spontaneous abduction of the small finger with weak intrinsics.

Physical examination revealed no acute swelling or erythema, and the skin was intact (Figure 1A). The patient was able to fully flex and extend his wrist, metacarpophalangeal joints, proximal interphalangeal joints, and distal interphalangeal joints in each digit. Loss of sensation was noted in the median nerve distribution with slight ulnar sensory loss in the small finger. The radial and ulnar artery pulses were within normal limits. Tinel’s test was positive at the carpal tunnel and Guyon’s canal and negative at the cubital tunnel.

After squeezing a stress ball for 10 to 15 seconds, there was acute swelling in the first web space and within the dorsal interossei and thenar musculature (Figure 1B). Complete loss of sensation in the ulnar nerve distribution as well as a positive Wartenberg’s sign (spontaneous unopposed abduction of the small finger) was demonstrated (Figure 1C). Repeat examination after 10 minutes of rest demonstrated decreased swelling, negative Wartenberg’s sign, and sensation in the ulnar nerve distribution returned to baseline.

Electromyography (EMG) showed findings consistent with carpal tunnel syndrome only. There was no evidence of cubital tunnel syndrome or signs of entrapment at Guyon’s canal. There was, however, abnormal motor and sensory amplitude and latency localized to the first dorsal interosseous muscle consistent with motor neuropathy. Magnetic resonance imaging (MRI) showed no signs of nerve pathology or soft tissue abnormalities. There were no masses that corresponded to the swelling in the first web space. The patient was diagnosed with carpal tunnel syndrome, clinical ulnar neuropathy, and chronic exertional compartment syndrome of the first dorsal interosseous and thenar compartments.

The patient was given the option of compartment pressure monitoring; however, he declined, and we did not feel these measurements would contribute significantly to the diagnosis as he was able to demonstrate these symptoms in the office on more than one occasion. Also, his physical examination was unchanged at each visit; he always showed acute swelling in the first web space, acute ulnar neuropathy, Wartenberg’s sign, and weakness of the thumb and hand.

**Figure 2:**
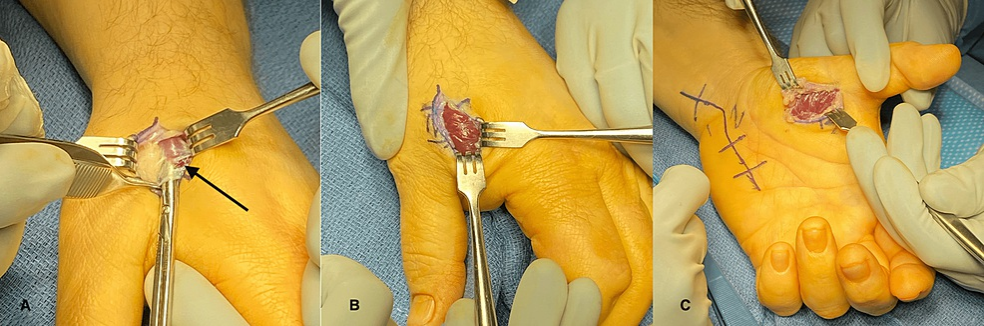
(A) Before fasciotomy. Muscle belly swelling and herniation is indicated by the arrowhead. (B) After fasciotomy. Notice the swollen appearance of the muscle belly. (C) Fasciotomy of the thenar compartment.

He was taken to the operating room for carpal tunnel release, Guyon’s canal release, and fasciotomies of the dorsal and palmar thumb. An incision was made in the first web space dorsally and careful dissection and release of the fascia overlying the first dorsal interossei musculature were performed. There was extreme tightness of the fascia and noticeable muscle swelling within the compartment with some muscle appearing to be herniating (Figure 2A, tip of arrowhead). The fascia was incised and the muscle immediately showed marked swelling (Figure 2B). A similar incision was made at the thenar compartment and the fascia was incised (Figure 2C). A Bruner-type incision was made over the volar ulnar wrist to release the ulnar nerve at the Guyon’s canal as well as the deep motor branch of the ulnar nerve distally. The carpal tunnel was accessed from this same incision and was subsequently released. No pathological findings of the nerve and no anatomic variants were appreciated. The patient’s final diagnosis following surgery was CECS involving the first dorsal and thenar compartment with concomitant acute ulnar neuropathy in the setting of carpal tunnel syndrome.

At the patient’s two-week post-operative follow-up appointment, the patient reported no pain or discomfort and noticed an improvement in numbness and tingling from his preoperative state. The patient did still experience some decreased strength and muscle fatigue subjectively, which he understood will take time to resolve. However, upon squeezing the stress ball, there was no reproducible swelling in the first dorsal interosseous or thenar compartment and his acute weakness was resolved. There Wartenberg’s sign was not present on exam. His subsequent post-operative appointments have shown continued gains in thumb and intrinsic strength. 

## Discussion

We present a rare case of CECS involving the first dorsal interosseous and thenar compartments of the hand with ipsilateral median and ulnar neuropathies. There are 10 compartments of the hand: four dorsal interosseous, three palmar interosseous, thenar, adductor, and hypothenar compartments [2]. The first dorsal interosseous compartment appears to be most commonly involved, presenting with pain and swelling in the first web space [2]. Other instances report thenar, hypothenar, and adductor compartment involvement, and rarely multiple compartments are involved at once [2]. Our case is unique in that it is the first report of simultaneous CECS in the hand with median and ulnar neuropathies. Providers should be aware of this rare presentation and recognize that a thorough physical examination can help to differentiate multiple hand conditions that present at the same time. This will spare the patient from complications that may arise from a missed diagnosis.

It is important to note that there were abnormal EMG findings at the first dorsal interosseous muscle that were indicative of motor neuropathy. While an abnormal EMG at this muscle could potentially make the diagnosis less clear, we believe that this motor neuropathy was caused by elevated compartment pressures compressing the ulnar-innervated muscle and impairing the nerve’s ability to fire effectively. These findings indicated that the patient had ongoing motor findings of the intrinsic hand muscles despite no obvious entrapment of the ulnar nerve at the wrist or elbow, further promoting our concern for exertional compartment syndrome in the setting of a generalized carpal tunnel diagnosis. In addition, the patient had an acute, reproducible Wartenberg’s sign, also indicating an exertional component to his acute hand weakness. It was our opinion that these clinical examination findings, as well as the abnormal nerve studies, were likely a result of elevated compartment pressure causally related to the patient’s exertion and occupation.

Furthermore, carpal tunnel syndrome would not create a Wartenberg’s sign, nor would it cause weakness of the dorsal intrinsic thumb muscles or swelling of the first web space with repetitive activity. This patient hence not only had exertional compartment syndrome of his first web space, but also a more acute type of ulnar nerve entrapment occurring at Guyon’s canal, creating small finger abduction deformity.

We recognize the literature states that measurement of the compartment pressure is the gold standard for confirming the diagnosis of CECS [4]. One of the major limitations of this case was that we did not collect compartment pressures before and after exertion. However, van den Brand et al. showed that compartment pressure measurement is only 77% sensitive [5]. Additionally, other literature suggests that compartment syndrome of the hand is largely a clinical diagnosis and measuring the compartment pressure is not necessary [6]. We were able to diagnose CECS with relative certainty through history and physical examination, and our treatment plan for this patient would not have changed if the compartment pressures were within the normal range. In addition, our patient was offered and declined the option of having his pressure taken. This seemed reasonable to us, as his symptoms were objectively reproducible on several occasions in the office with photographs taken of acute thenar swelling and a positive Wartenberg’s sign. It was our belief that his patient needed an elective fasciotomy regardless of the compartment pressure. The patient was agreeable to not have them checked, and the postsurgical resolution of his symptoms further supports our diagnosis and the causality of the patient’s hand dysfunction regarding CECS.

## Conclusions

CECS of the hand is a rare condition, and to our knowledge, this is the first report of hand CECS occurring with median and ulnar neuropathies at the same time. Diagnosis can typically be achieved by detailed history and physical examination. Measuring the compartment pressure can be used to confirm the diagnosis, but is not necessary, especially if the results will not change the treatment plan. Elective fasciotomy with carpal tunnel and ulnar nerve releases was curative in our patient. We attest to the causal relationship of this patient’s CECS and his thenar swelling, weakness, and acute ulnar nerve findings, and conclude that it is important for providers to perform a thorough physical examination of the hand to differentiate multiple hand conditions that may present at the same time, and not rely only on EMG, MRI, or radiograph, but also on clinical examination skills, knowledge of anatomy, and pure observation.
